# Supplementary material for: CSF Proteomics and Machine Learning Reveal Distinct Stages Across the Alzheimer’s Disease Continuum
Source: medRxiv. 2025 Nov 13:2025.11.05.25339138. Preprint. [Version 1] doi: 10.1101/2025.11.05.25339138 (PMC12642759; doi:10.1101/2025.11.05.25339138)
Supplement: 1 [file NIHPP2025.11.05.25339138V1-supplement-1.pdf]

List of Supplementary Materials

fig. S1. Quality control of the ADNI CSF proteome. (A) Multidimensional scaling (MDS) illustrating TMT-MS batch correction. Log2 abundance, log2 abundance divided by the global internal standard (GIS), and TAMPOR are shown. (B) Variance partition plots were used to visualize the percent variance of each protein in the dataset co-varying with batch, age and sex. The matrix was subjected to bootstrap regression (right) to remove variance due to batch.

fig. S2. **MS proteomics predicts diagnostic groups in ADNI. (A-B)** MS proteomics data were used to train a classification model. ROC curves from 100 permuted runs illustrate the median Area Under the Curve (AUC) based on an 80–20% train-test split for the following comparisons: (A) AD Dementia/MCI (due-to-AD) versus AT- Controls (AUC=0.97), and (B) asymptomatic AD versus AT- Controls (AUC=0.91). Higher AUC values indicate better classification performance,

with values closer to 1.0 reflecting greater sensitivity and specificity. **(C-D)** Top SHAP feature contributions corresponding to the median AUC models shown in A and B are visualized for: (C) AD Dementia/MCI (due-to-AD) versus AT- Controls, and (D) asymptomatic AD versus AT- Controls.

fig. S3. **MS proteomics estimates baseline clinical diagnosis in ADNI. (A-C)** Differential protein expression across diagnostic groups is shown. Volcano plots depict differentially abundant proteins (DAPs) when comparing individuals across diagnostic categories (Controls = 244, MCI = 563, AD = 164): (A) MCI versus Controls, (B) AD versus MCI, and (C) AD versus Controls. Proteins with $P_{FDR}<0.05$ (marked by red line) were considered significant. For clarity, top selected proteins are labeled. **(D)** A heatmap of Pearson correlations is shown for MS proteomics and clinical diagnosis. The MS proteins are labeled as their respective gene symbols, and the strength and direction of correlation is shown by the blue to green color scale. The top 30 proteins with the strongest correlations were selected from each category, and their union is displayed in the heatmaps. **(E-G)** MS proteomics data were used to train a classification model. ROC curves from 100 permuted runs illustrate the median Area Under the Curve (AUC) based on an 80–20% train-test split for the following comparisons: (E) MCI vs. Controls (AUC = 0.82), (F) AD vs. MCI (AUC = 0.90), and (G) AD vs. Controls (AUC = 0.99). Higher AUC values indicate better classification performance, with values closer to 1.0 reflecting greater sensitivity and specificity. **(H-J)** Top SHAP feature contributions corresponding to the median AUC models shown in panels (E-G) are visualized for: (H) MCI vs. Controls, (I) AD vs. MCI, and (J) AD vs. Controls. Colors are assigned to individual proteins names based on their assignment in the brain-derived modules (12).

fig. S4. **Comparison of AChE expression levels in ADNI participants based on treatment status.** Participants treated with Donepezil, Galantamine, Rivastigmine, or Memantine (n=53) and non-treated participants were included in this analysis**. (A)** AChE levels compared between participants treated with cholinergic medications compared to untreated participants prior to CSF collection ($P=1.16\times10^{-9}$). **(B)** AChE levels compared between controls and MCI/AD participants regardless of the treatment status ($P=2.2\times10^{-19}$). **(C)** AChE levels between treated versus non-treated AD and MCI participants ($P=2.52\times10^{-5}$).

fig. S5. **Summary of functional enrichment analyses using Gene Ontology (GO) databases for different categories of proteins.** Biological pathways that were up- or downregulated in association with neuroimaging-based biomarkers of **(A-B)** neurodegeneration (hippocampal volume and FDG-PET SUVR) and **(C-D)** pathological burden (tau-PET neocortical SUVR and Aβ-PET Centiloid value) are shown.

table S1. Traits of 1,104 participants from the ADNI cohort.
table S2. Demographic and clinical characteristics of study participants from the ADNI cohort.
table S3. Differential abundance analysis of CSF proteins associated with diagnostic groups (TMT-MS).
table S4. Differential abundance analysis of CSF proteins shared between the targeted Selected Reaction Monitoring (SRM) panel and the TMT-MS proteomics cohort across diagnostic groups.
table S5. Differential abundance analysis of CSF proteins associated with diagnostic groups (SomaLogic).

table S6. Classification performance across 100 iterations of random subsampling to distinguish AT- controls from participants with AD pathology [MCI (due-to-AD) and AD Dementia] using TMT-MS proteomics.

table S7. Differential abundance analysis of proteins (using TMT-MS) previously identified as altered along the disease timeline in the DIAN-TU autosomal dominant Alzheimer's disease cohort.

table S8. Classification performance across 100 iterations of random subsampling to distinguish AT- controls from asymptomatic AD using TMT-MS proteomics.

table S9. Differential abundance analysis of CSF proteins across Alzheimer's disease stages (TMT-MS).

table S10. Protein panels selected by machine learning models to distinguish AD stages using least absolute shrinkage and selection operator (LASSO).

table S11. Differential abundance analysis of TMT-MS proteins correlated with baseline clinical measures of cognition and dementia severity.

table S12. Differential abundance analysis of TMT-MS proteins correlated with baseline neuroimaging biomarkers of neurodegeneration and pathology.

Supplementary Material

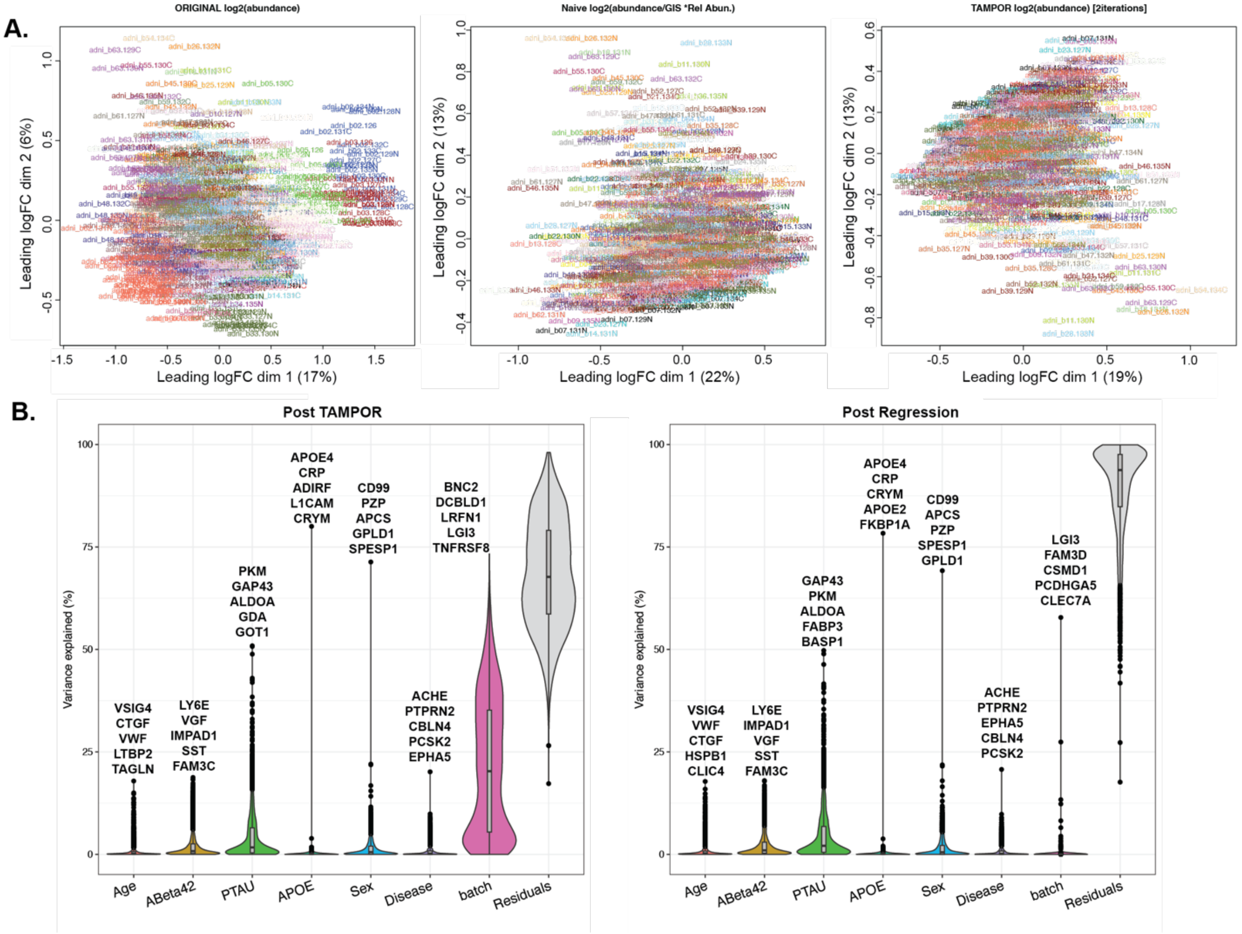

fig. S1. Quality control of the ADNI CSF proteome. (A) Multidimensional scaling (MDS) illustrating TMT-MS batch correction. Log2 abundance, log2 abundance divided by the global internal standard (GIS), and TAMPOR are shown. (B) Variance partition plots were used to visualize the percent variance of each protein in the dataset co-varying with batch, age and sex. The matrix was subjected to bootstrap regression (right) to remove variance due to batch.

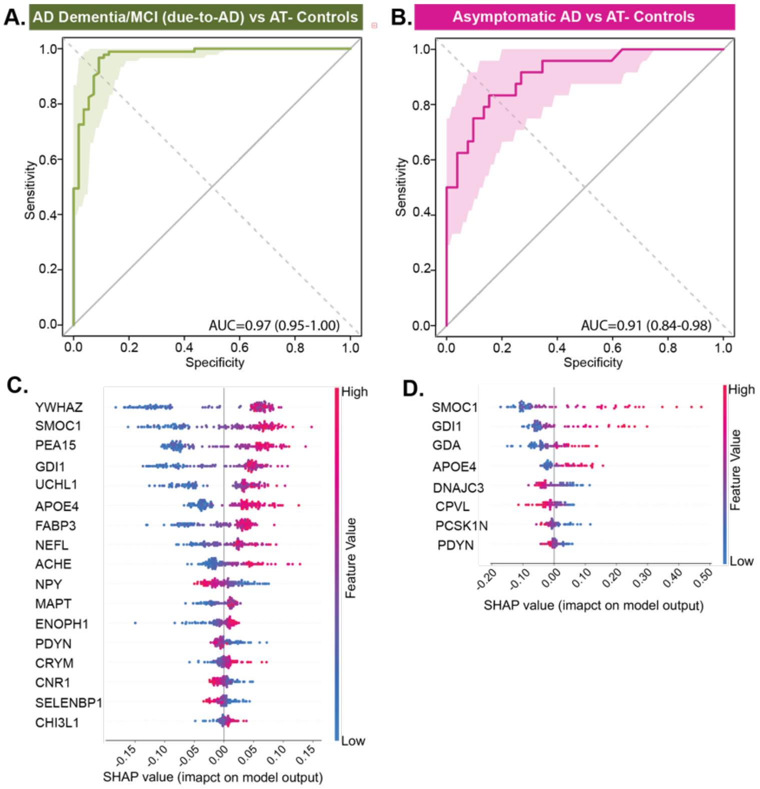

fig. S2. **MS proteomics predicts diagnostic groups in ADNI. (A-B)** MS proteomics data were used to train a classification model. ROC curves from 100 permuted runs illustrate the median Area Under the Curve (AUC) based on an 80–20% train-test split for the following comparisons: (A) AD Dementia/MCI (due-to-AD) versus AT- Controls (AUC=0.97), and (B) asymptomatic AD versus AT- Controls (AUC=0.91). Higher AUC values indicate better classification performance, with values closer to 1.0 reflecting greater sensitivity and specificity. **(C-D)** Top SHAP feature contributions corresponding to the median AUC models shown in A and B are visualized for: (C) AD Dementia/MCI (due-to-AD) versus AT- Controls, and (D) asymptomatic AD versus AT- Controls.

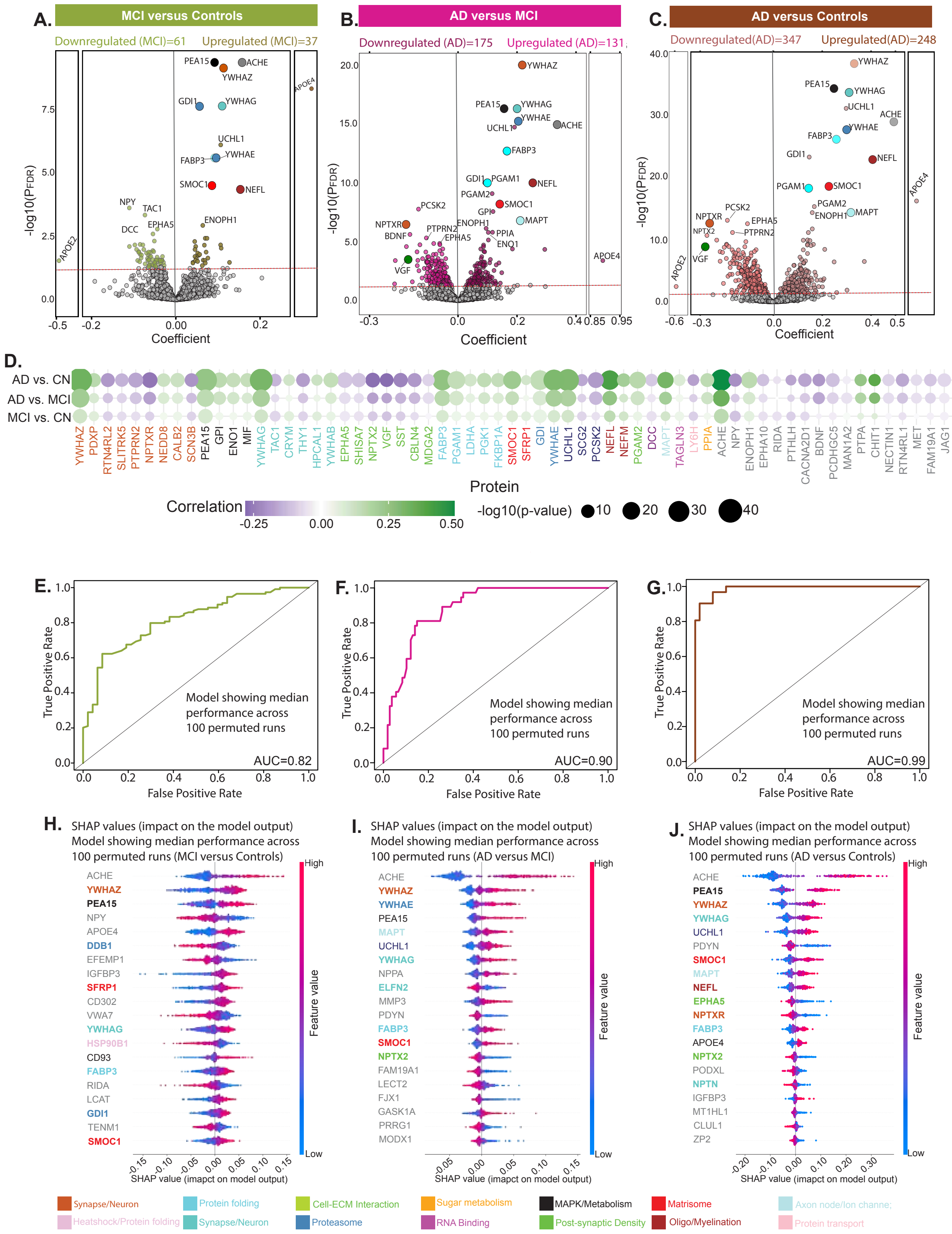

fig. S3. **MS proteomics estimates baseline clinical diagnosis in ADNI. (A-C)** Differential protein expression across diagnostic groups is shown. Volcano plots depict differentially

abundant proteins (DAPs) when comparing individuals across diagnostic categories (Controls = 244, MCI = 563, AD = 164): (A) MCI versus Controls, (B) AD versus MCI, and (C) AD versus Controls. Proteins with $P_{FDR}<0.05$ (marked by red line) were considered significant. For clarity, top selected proteins are labeled. **(D)** A heatmap of Pearson correlations is shown for MS proteomics and clinical diagnosis. The MS proteins are labeled as their respective gene symbols, and the strength and direction of correlation is shown by the blue to green color scale. The top 30 proteins with the strongest correlations were selected from each category, and their union is displayed in the heatmaps. **(E-G)** MS proteomics data were used to train a classification model. ROC curves from 100 permuted runs illustrate the median Area Under the Curve (AUC) based on an 80–20% train-test split for the following comparisons: (E) MCI vs. Controls (AUC = 0.82), (F) AD vs. MCI (AUC = 0.90), and (G) AD vs. Controls (AUC = 0.99). Higher AUC values indicate better classification performance, with values closer to 1.0 reflecting greater sensitivity and specificity. **(H-J)** Top SHAP feature contributions corresponding to the median AUC models shown in panels (E-G) are visualized for: (H) MCI vs. Controls, (I) AD vs. MCI, and (J) AD vs. Controls. Colors are assigned to individual proteins names based on their assignment in the brain-derived modules (12).

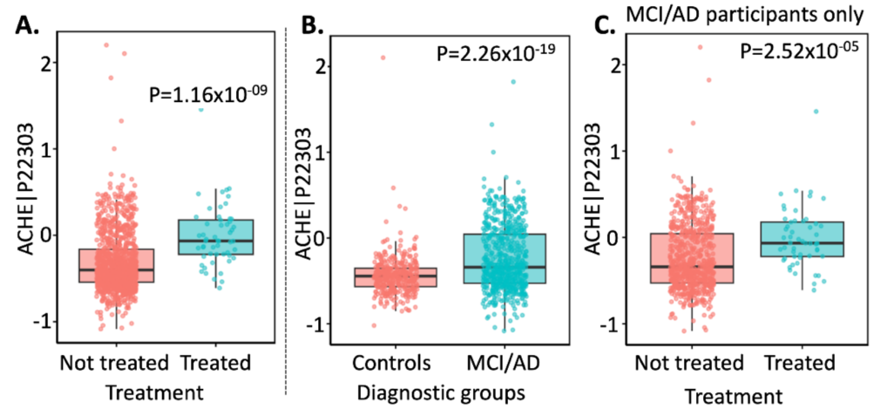

fig. S4. **Comparison of AChE expression levels in ADNI participants based on treatment status.** Participants treated with Donepezil, Galantamine, Rivastigmine, or Memantine (n=53) and non-treated participants were included in this analysis**. (A)** AChE levels compared between participants treated with cholinergic medications compared to untreated participants prior to CSF collection ($P=1.16\times10^{-9}$). **(B)** AChE levels compared between controls and MCI/AD participants regardless of the treatment status ($P=2.2\times10^{-19}$). **(C)** AChE levels between treated versus non-treated AD and MCI participants ($P=2.52\times10^{-5}$).

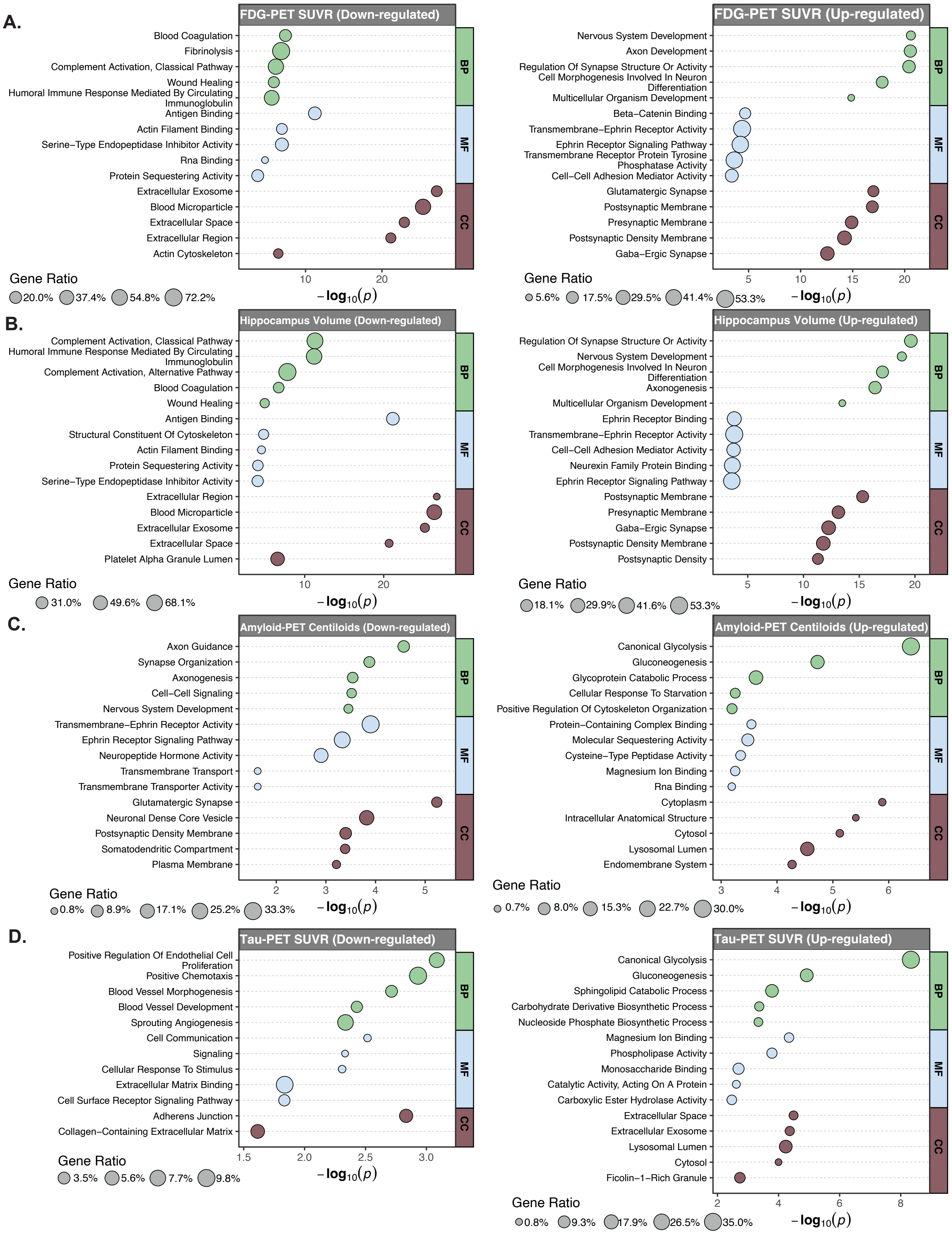

fig. S5. Summary of functional enrichment analyses using Gene Ontology (GO) databases for different categories of proteins. Biological pathways that were up- or downregulated

in association with neuroimaging-based biomarkers of **(A-B)** neurodegeneration (hippocampal volume and FDG-PET SUVR) and **(C-D)** pathological burden (tau-PET neocortical SUVR and Aβ-PET Centiloid value) are shown.
